# Graphitic Carbon Nitride‐Based Microrobots: Progress and Prospects in Environmental and Biomedical Applications

**DOI:** 10.1002/smll.75905

**Published:** 2026-10-06

**Authors:** Yunhuan Yuan, Martin Pumera

**Affiliations:** ^1^ Future Energy and Innovation Laboratory Central European Institute of Technology Brno University of Technology Brno Czech Republic; ^2^ Department of Medical Research China Medical University Hospital China Medical University Taichung Taiwan; ^3^ Office of Research and Development Asia University Taichung Taiwan; ^4^ Advanced Nanorobots & Multiscale Robotics Laboratory Faculty of Electrical Engineering and Computer Science VSB – Technical University of Ostrava Ostrava Czech Republic; ^5^ Department of Chemical and Biomolecular Engineering Yonsei University Seodaemun‐gu Seoul South Korea

**Keywords:** biomedical applications, environmental remediation, graphitic carbon nitride, micromotors, photocatalysis, self‐propulsion

## Abstract

Graphitic carbon nitride (g‐C_3_N_4_) is a metal‐free semiconductor responsive to visible light that has attracted considerable interest as a photocatalyst in environmental and biomedical applications. Constructing photocatalysts as light‐driven microrobots allows simultaneous propulsion and catalytic activity, both driven by photoinduced charge separation. In addition, light‐driven systems can perform remote control of micro/nanorobots (MNRs) and reduce their reliance on external chemical fuels. Thus, g‐C_3_N_4_ has been investigated as a functional material for microrobots, owing to its low cost, chemical stability, and biocompatibility. This review presents an overview of g‐C_3_N_4_‐based microrobots, including their fabrication strategies for different morphologies, such as tubular, sheet‐like, and spherical structures. It also discusses their propulsion mechanisms, including self‐diffusiophoresis, self‐electrophoresis, and bubble propulsion. Their applications in water purification, including the degradation of organic pollutants and the removal of heavy metals, are discussed, followed by biomedical uses such as antibacterial therapy and drug delivery. Finally, current challenges and prospects for the development of efficient and sustainable g‐C_3_N_4_‐based microrobots are outlined.

## Graphical abstract

This Review summarizes recent progress in g‐C_3_N_4_‐based microrobots, including fabrication strategies for different morphologies and their main propulsion mechanisms. Their applications in environmental remediation and biomedicine, including water purification, antibacterial therapy, and drug delivery, are discussed. The key challenges and possible strategies for developing efficient and sustainable g‐C_3_N_4_‐based microrobots are also highlighted.

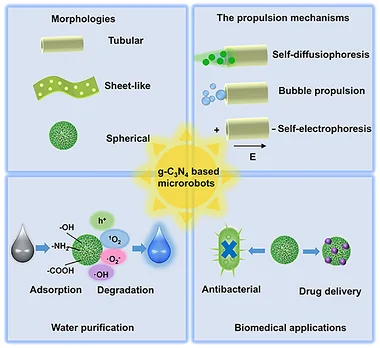

## 1 Introduction

Micro/nanorobots (MNRs) have emerged as a rapidly developing class of intelligent devices that harvest energy from diverse physical or chemical sources in the surrounding environment to perform autonomous motion. In this review, the term “micro/nanorobots (MNRs)” is used broadly to include both micro/nanomotors and micro/nanoswimmers. Owing to their small size, autonomous motion, and task‐specific design, MNRs have been applied in environmental remediation and biomedical fields [1, 2, 3, 4, 5]. A variety of propulsion methods have been developed, including the use of chemical fuels, magnetic fields, ultrasound, light, and electric fields [6, 7]. Chemical propulsion usually requires a high concentration of fuel to achieve strong propulsion, which can be harmful to both biological systems and the environment [8]. Magnetic field propulsion depends on complex external equipment for magnetic field generation and control, which limits its real‐world application [9]. Ultrasound‐based propulsion methods suffer from low energy conversion efficiency [10]. Electric field‐driven microrobots rely on the electrical conductivity of the surrounding medium, making them difficult to apply in physiological environments [11].

Light is one of the most widely used external stimuli, with common material types including photocatalytic [2, 7, 12], photothermal [13], photochromic [14], photolytic [15], and photoisomerizable systems [16]. Among light‐driven MNRs, photocatalytic and photoinduced thermophoretic propulsion have attracted growing interest due to their superior performance compared to other strategies [1, 6]. Thermophoretic MNRs are driven by local temperature gradients but require high‐intensity light to induce a significant temperature increase [1, 6]. Photocatalytic MNRs, which are easy to synthesize and can efficiently convert light and chemical energy into mechanical motion, have been extensively applied in the fabrication of light‐driven MNRs [1, 6]. Moreover, owing to their distinct absorption properties, photocatalytic materials can efficiently utilize light across a broad spectrum from ultraviolet to visible wavelengths [17]. Upon light irradiation, photonic energy excites electrons from the valence band to the conduction band of the material, thereby generating electron–hole pairs that initiate redox reactions with chemical species in the surrounding liquid medium [1, 2, 18]. To date, the movement mechanisms of photocatalytic MNRs include light‐induced self‐diffusiophoretic propulsion, self‐electrophoretic propulsion, and bubble propulsion [1]. Light‐induced self‐diffusiophoretic propulsion refers to the process in which photocatalytic reactions generate electrolytic and non‐electrolytic species, creating chemical concentration gradients that drive the motion of MNRs in a liquid medium [6], such as a TiO_2_–SiO_2_ mesoporous carbon nanomotor under UV illumination [19]. Self‐electrophoretic propulsion is achieved through a self‐generated electric field (E) created by the asymmetric distribution of ions around the microrobots [6], like the BiVO_4_ microparticles fabricated by Heckel et al. [20]. Bubble propulsion occurs when materials react with the surrounding medium under light, generating a stream of bubbles that enables the rapid movement of MNRs [6]. Currently, photocatalysts, such as TiO_2_, ZnO, Bi‐based materials, Cu_2_O, Ag_3_PO_4_, and Fe_2_O_3_, have been applied in the fabrication of photocatalytic MNRs [21, 22, 23]. Among these, TiO_2_‐based materials are the most common, but they usually require intense ultraviolet light or the incorporation of precious metals to achieve strong propulsion [24]. Although Bi‐based materials, Cu_2_O, and Ag_3_PO_4_ can move under visible light, they are costly and contain large amounts of metallic elements, which limit their environmental and biological compatibility.

Graphitic carbon nitride (g‐C_3_N_4_) is considered an effective photocatalyst owing to its ability to absorb light across the ultraviolet–visible spectrum. Moreover, its low cost, chemical stability, biocompatibility, and environmental friendliness make it a promising candidate for microrobot applications in environmental remediation and biomedicine. In recent years, a variety of fabrication strategies for g‐C_3_N_4_‐based microrobots have been studied, along with investigations into their propulsion behaviors. Most studies have focused on applications in pollutant removal [2, 25, 26], while only limited efforts have explored their biomedical applications [27]. In this review, we summarize the fabrication methods, propulsion mechanisms, and applications of g‐C_3_N_4_‐based microrobots in water purification and biomedicine, and discuss the key challenges and future directions (Figure 1). The review compares fabrication strategies for tubular, sheet‐like, and spherical g‐C_3_N_4_‐based microrobots and incorporates recent developments in the field. In addition, the challenges that may limit the future development of g‐C_3_N_4_‐based microrobots in environmental and biomedical applications are discussed, together with possible strategies to address these problems. For biomedical applications, the discussion covers challenges such as biosafety, biodegradability, in vivo behavior, and limited light penetration, as well as potential strategies and recent advances relevant to future development.

**FIGURE 1:**
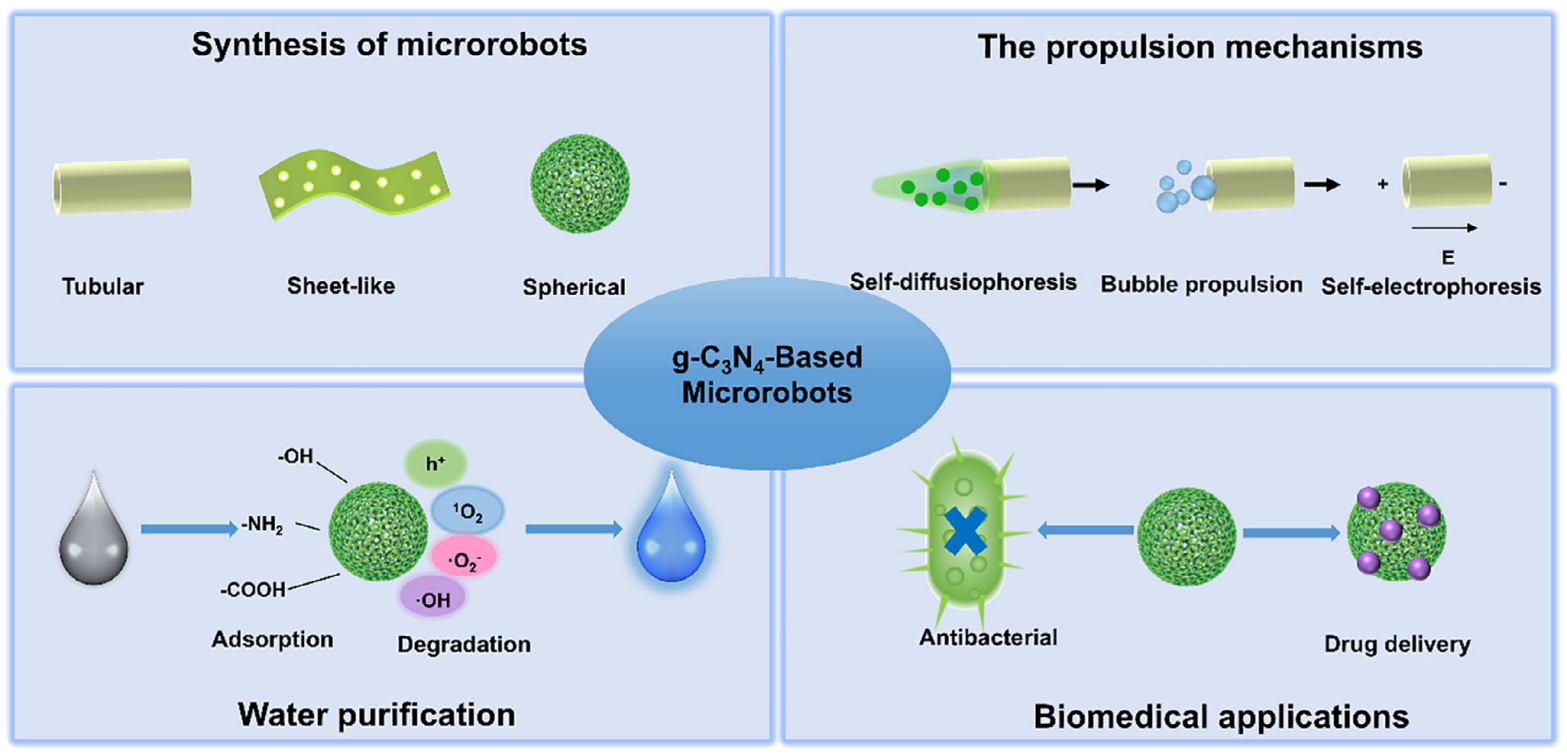
Schematic illustration of the representative morphologies, propulsion mechanisms, and main applications of g‐C_3_N_4_‐based microrobots in water purification and biomedical fields.

## 2 Synthesis of g‐C_3_N_4_‐Based Microrobots

g‐C_3_N_4_ is typically prepared through thermal condensation of low‐cost, nitrogen‐rich precursors, such as melamine, urea, thiourea, and cyanamide [28]. The morphology of g‐C_3_N_4_‐based microrobots usually includes tubular, sheet‐like, and spherical structures. Tubular g‐C_3_N_4_ is generally prepared using a template‐free approach based on melamine–cyanuric acid supramolecular (MCS) assemblies, followed by thermal calcination to form 1D hollow structures. Sheet‐like g‐C_3_N_4_ is commonly produced by directly calcining nitrogen‐rich compounds in air, such as melamine or urea. Several methods have been employed to fabricate spherical g‐C_3_N_4_‐based structures for microrobots, including emulsion‐based techniques, mixing followed by hydrothermal and annealing treatments, co‐calcination, and solvothermal synthesis. The choice of preparation method depends on the desired morphology of g‐C_3_N_4_. The fabrication strategies for microrobots with different g‐C_3_N_4_ morphologies are discussed in the following sections (Figure 2).

**FIGURE 2:**
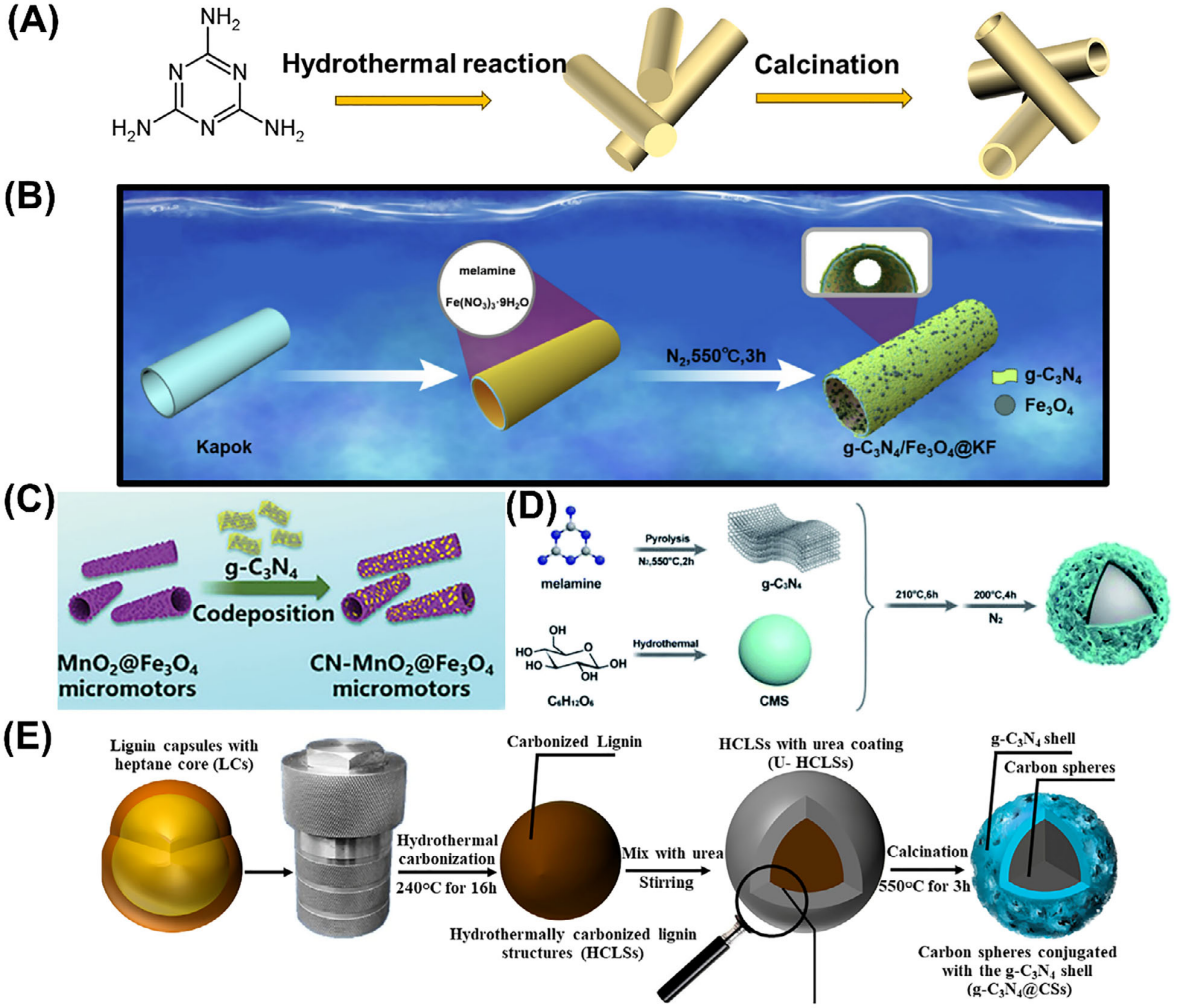
Fabrication schemes for g‐C_3_N_4_ microrobots with different morphologies. (A) Tubular g‐C_3_N_4_ microrobots. Reproduced with permission [2]. under the CC BY 4.0 license. Copyright 2025, The Authors. (B) Tubular g‐C_3_N_4_–Fe_3_O_4_@KF microrobots. Reproduced with permission [29]. Copyright 2022, Elsevier. (C) CN–MnO_2_@Fe_3_O_4_ microrobots. Reproduced with permission [30]. Copyright 2023, Wiley‐VCH. (D) g‐C_3_N_4_@carbon microsphere. Reproduced with permission [31]. from the Royal Society of Chemistry. Copyright 2022, The Authors. (E) g‐C_3_N_4_@carbon spheres. Reproduced with permission [32]. under the CC BY 4.0 license. Copyright 2021, The Authors.

To fabricate tubular microrobots, our group reported the synthesis of g‐C_3_N_4_ microtubes using a template‐free approach (Figure 2A) [2]. Precursor nanorods were first obtained through the hydrolysis of melamine and subsequent supramolecular self‐assembly. The resulting nanorods were then transferred to a tube furnace and calcined at 500°C under an N_2_ atmosphere to form 1D hollow g‐C_3_N_4_ microtubes. The apparent optical bandgap (Eg) was estimated to be 2.6 eV from the UV–vis diffuse reflectance spectrum using the Tauc method. This value is lower than that typically reported for bulk g‐C_3_N_4_. This lower apparent value may be related to enhanced multiple scattering of light within the microtube structure [2]. Then, a 15 nm thick Ag layer was coated onto hollow g‐C_3_N_4_ microtubes to construct g‐C_3_N_4_/Ag Janus microrobots, with Ag in the metallic state (Ag^0^) [33]. Electrochemical impedance spectroscopy and photocurrent measurements showed that Ag introduction facilitates charge generation and separation, inhibits recombination, and ultimately enhances the photocatalytic performance of the g‐C_3_N_4_/Ag system. Additionally, our group reported another template‐free synthesis of g‐C_3_N_4_ microrobots [34]. Self‐assembled intermediate microrods were first obtained through a hydrothermal reaction between melamine and H_3_PO_4_, followed by calcination at 500°C for 4 h to obtain tubular microrobots. The resulting structures had an average diameter of 9.7 µm and an average length of 67 µm, and were composed of carbon and nitrogen.

Template‐based strategies have also been employed to fabricate tubular microrobots. For example, Zheng et al. applied kapok fiber (KF) as a template [29]. A mixture of melamine and ferric nitrate nonahydrate was hydrothermally treated, leading to deposition of g‐C_3_N_4_ and Fe_3_O_4_ on both the inner and outer surfaces of the KF. After drying, the above sample was calcined under an N_2_ atmosphere to form g‐C_3_N_4_/Fe_3_O_4_‐coated tubular microrobots (Figure 2B). XRD results show that the g‐C_3_N_4_–Fe_3_O_4_@KF tubular structures display characteristic diffraction peaks from both g‐C_3_N_4_ and Fe_3_O_4_. The Fe_3_O_4_ signals are relatively weak. This may indicate interactions between the two components and reduced crystallinity of the composite. All observed peaks correspond to g‐C_3_N_4_ or Fe_3_O_4_, with no detectable impurity phases. EDX and XPS analyses also confirm the presence of carbon, nitrogen, oxygen, and iron in the structure.

Dong et al. reported the preparation of sheet‐like g‐C_3_N_4_ by heating urea at 520°C, followed by thermal oxidation etching at 500°C to obtain the structure. The product was then combined with carbon black (CB) through sonication and low‐temperature heating to form CB/g‐C_3_N_4_ composites [35]. In another study, melamine was directly heated at 600°C for 2 h to obtain bulk g‐C_3_N_4_. The product was further treated by ultrasonication for 10 h to acquire g‐C_3_N_4_ nanosheets [30]. These nanosheets were mixed with MnO_2_@Fe_3_O_4_ and were shaken overnight, then centrifuged, washed, and vacuum‐dried to produce CN‐MnO_2_@Fe_3_O_4_ microrobots (Figure 2C).

Ye et al. transformed g‐C_3_N_4_ sheets into spherical structures using an emulsion method [36]. They first prepared g‐C_3_N_4_ nanosheets by thermal condensation of urea followed by 12 h of ultrasonication and subsequent washing. Platinum nanoparticles (PtNPs) were then grown on the surface of the nanosheets using an in situ synthesis method to form Pt–g‐C_3_N_4_ nanosheets. Spherical Pt–g‐C_3_N_4_ particles were subsequently fabricated through the emulsion method. Specifically, a 5 wt% polyvinylpyrrolidone (PVP) solution was added to the Pt–g‐C_3_N_4_ nanosheet suspension, after which the suspension (1 mL) was blended with 30 mL of olive oil in the presence of 1 mg of Span 80. After stirring for 15 min, the mixture was heated at 90°C for 2 h. The resulting spherical Pt–g‐C_3_N_4_ particles, with an average diameter of approximately 2.6 µm, were collected by centrifugation. To prepare spherical Pt–g‐C_3_N_4_/Fe_3_O_4_ particles, 0.2 mL of Fe_3_O_4_ nanoparticle suspension was added to the PVP‐containing Pt–g‐C_3_N_4_ nanosheet solution before mixing with olive oil. NH_2_–Pt–g‐C_3_N_4_ micromotors were prepared in the final step by immersing the particles in 2‐mercaptoethylamine for 24 h. Tong et al. developed a similar method using an emulsion solvent evaporation strategy to fabricate carbon nitride (C_3_N_4_)/polypyrrole nanoparticle (PPyNP)‐based microrobots [37]. g‐C_3_N_4_ nanosheets were first synthesized by thermal condensation of urea, followed by ultrasonication in methanol and solvent evaporation. Polypyrrole nanoparticles (PPyNPs) were produced through chemical polymerization in the presence of FeCl_3_ and PVA. A 1:1 mixture of g‐C_3_N_4_ and PPyNPs was dispersed in gelatin solution and emulsified in liquid paraffin containing Span 80, forming spherical structures that were subsequently stabilized by glutaraldehyde crosslinking.

The g‐C_3_N_4_@carbon microspheres (CMSs) were prepared by mixing followed by hydrothermal and annealing treatments [31]. First, melamine was pyrolyzed at 550°C for 2 h in a muffle furnace to obtain g‐C_3_N_4_, while CMSs were synthesized through a hydrothermal reaction using glucose and cetyltrimethylammonium bromide. The g‐C_3_N_4_ and CMSs were then mixed and subjected to subsequent hydrothermal and annealing treatments to form g‐C_3_N_4_@CMSs. The CMSs are fully encapsulated by 2D g‐C_3_N_4_ sheets, forming a tightly packed shell around the microsphere core (Figure 2D). The resulting g‐C_3_N_4_@CMS microrobots have a diameter of approximately 13 µm and contain numerous micropores. Similarly, Cui et al. fabricated g‐C_3_N_4_@carbon spheres (CSs) through a co‐calcination strategy, in which hydrothermally carbonized lignin spheres were mixed with urea and calcined at 550°C for 3 h, resulting in composite spheres with an average diameter of around 0.7 µm (Figure 2E) [32]. Subsequently, a 10 nm Pt/Pd layer was deposited on one side of the composite spheres through sputter coating. Notably, hydroxyl groups on the surface of the CSs can form hydrogen bonds with urea molecules, which facilitate the uniform coating of g‐C_3_N_4_ onto the carbon spheres.

Janus Fe/C_3_N_4_ microrobots, with an average diameter of about 1 µm, were fabricated through a solvothermal reaction and subsequent sputter coating [38]. Spherical C_3_N_4_ was first obtained through a solvothermal reaction of cyanuric chloride in acetonitrile at 200 °C. After ultrasonic treatment in ethanol to achieve uniform dispersion, the C_3_N_4_ spheres were spread onto a glass slide. A 50 nm thick iron layer was then deposited by sputter coating, resulting in the formation of Janus Fe/C_3_N_4_ microrobots. XPS analysis shows Fe 2p peaks at 717.8 eV and 730.0 eV, which are slightly higher than commonly reported values (∼711 eV and ∼725 eV), suggesting the possible formation of iron nitrides. The weak diffraction peaks at 35.3° and 56.1° in the XRD pattern correspond to Fe.

Overall, the preparation methods of g‐C_3_N_4_ depend on the desired morphology. Tubular g‐C_3_N_4_ structures can be fabricated through both template‐free and template‐based routes, while sheet‐like g‐C_3_N_4_ is generally combined with other materials to obtain specific functions, and spherical g‐C_3_N_4_‐based MNRs can be produced through several different strategies. From a fabrication perspective, sheet‐like structures are relatively easy to prepare through direct thermal treatment, although additional components are generally required to achieve effective propulsion or targeted functions. In contrast, tubular and spherical g‐C_3_N_4_ structures can be directly fabricated in the desired morphology without necessarily requiring additional materials, although their synthesis usually involves more processing steps and more complex procedures.

## 3 The Propulsion Mechanisms of g‐C_3_N_4_‐Based Microrobots

The propulsion of photocatalytic MNRs is primarily driven by chemical gradients or asymmetric distributions of photocatalytic products generated during light‐induced reactions [1, 3]. g‐C_3_N_4_ is one of the representative photocatalytic materials [2]. Under light irradiation at a specific wavelength, charge carriers are generated and separated. Electrons are excited from the valence band (VB) to the conduction band (CB) while holes remain in the valence band. The photogenerated electrons and holes then react with surrounding species, producing molecules, ions, or gases. As a result, chemical gradients, uneven ion distributions, or bubble formation can drive the motion of the MNRs. This review focuses on g‐C_3_N_4_‐based propulsion mechanisms, including self‐diffusiophoretic propulsion, bubble propulsion, and self‐electrophoretic propulsion (Table 1). Here, “fuel‐free” refers to propulsion in water without externally added chemical fuels such as H_2_O_2_ or methanol. Systems in which g‐C_3_N_4_ acts only as a photocatalyst for pollutant degradation or fluorescence sensing are not discussed in detail.

**TABLE 1:** Propulsion mechanisms, speeds, and applications of graphitic carbon nitride‐based microrobots.

**Microrobot**	**Propulsion Mechanism**	**Wavelength (nm)**	**Fuel**	**Maximum Speed (S) or Diffusion (D)**	**Application**	**Refs**.
g‐C_3_N_4_ (Microtube)	Self‐diffusiophoresis	365 and 400	1.5 wt% H_2_O_2_	S = 6.9 µm/s	Tetracycline degradation	2
g‐C_3_N_4_/Ag Janus (Microtube)	Self‐diffusiophoresis	365	1 wt% H_2_O_2_	S = 7 µm/s	Tetracycline degradation	33
Fe_3_O_4_/f‐C_3_N_4_ (Sphere)	Self‐diffusiophoresis	Visible light	0.2 wt% H_2_O_2_	D = 1.77 µm^2^/s	Tetracycline degradation	25
Pt‐g‐C_3_N_4_ (Sphere)	Self‐diffusiophoresis	White light	Fuel‐free	S = 23 µm/s	No	36
PHI‐Au,Pt, SiO_2_	Self‐diffusiophoresis, self‐electrophoresis	365	Fuel‐free 5 vol% methanol 0.5 vol% H_2_O_2_	S = 10.4 µm/s S = 20.4 µm/s S = 24.6 µm/s	No	39
C_3_N_4_/PPyNP‐based (Sphere)	self‐diffusiophoresis, self‐thermophoresis	Visible light	Fuel‐free	S = 5.5 µm/s S = 8 µm/s	No	37
g‐C_3_N_4_ (Tubular)	Bubble propulsion	Visible light	20 wt% H_2_O_2_	S = 72 µm/s	Removal of heavy metals	34
g‐C_3_N_4_‐Fe_3_O_4_@KF (Tubular)	Bubble propulsion	420	30 wt% H_2_O_2_	S = 178.9 µm/s	Rhodamine B degradation	29
Pt/Pd@g‐C_3_N_4_@CSs (Sphere)	Bubble propulsion	Visible light	7.5% H_2_O_2_	S = 14.5 µm/s	Rhodamine B degradation	32
g‐C_3_N_4_@CMS(Sphere)	Bubble propulsion	420	30 wt% H_2_O_2_	S = 167.97 µm/s	Rhodamine B degradation	31
Fe/C_3_N_4_ (Sphere)	Self‐electrophoresis, liquid‐phase bubble generation	532 nm laser beam	0.5 vol% H_2_O_2_ with 5 ppm Cr (VI)	D = 44 ×10^6^ nm^2^/s	Sulfamethaxazole degradation	38

Tubular g‐C_3_N_4_ microrobots are driven by self‐diffusiophoretic propulsion resulting from the asymmetric distribution of chemical species around the microrobots [2]. It is notable that their motion can be programmed by the illumination wavelength (Figure 3A,B). Under 365 nm illumination from below, the microrobots moved upward and showed negative photogravitaxis as the combined effects of light‐driven propulsion and buoyancy overcame gravity (Figure 3A). However, under 400 nm illumination, some microrobots showed 2D motion while others moved in three dimensions but remained within the field of view (Figure 3B). Then, we explored the propulsion mechanism using electron paramagnetic resonance (EPR) together with motion experiments under different illumination directions (bottom and left). EPR results indicate that under light irradiation, the microrobots facilitate photocatalytic redox processes in the surrounding medium, generating reactive species such as •O_2_^−^, •OH, and ^1^O_2_. This leads to an asymmetric distribution of reaction products, forming chemical gradients that drive self‐diffusiophoretic propulsion. When illuminated vertically from the bottom, photogenerated charge carriers tend to accumulate at the base of the microrobots, which enhances local redox reactions and strengthens the chemical gradient. Together with buoyancy, the resulting propulsion force effectively counteracts gravity, leading to negative photogravitactic motion. In contrast, when illuminated from the left side, charge carriers do not accumulate at the base, resulting in a weaker vertical chemical gradient and insufficient upward propulsion to overcome gravity. In addition, the g‐C_3_N_4_/Ag Janus microrobots display a similar self‐diffusiophoretic propulsion mechanism and exhibit negative photogravitactic motion [33].

**FIGURE 3:**
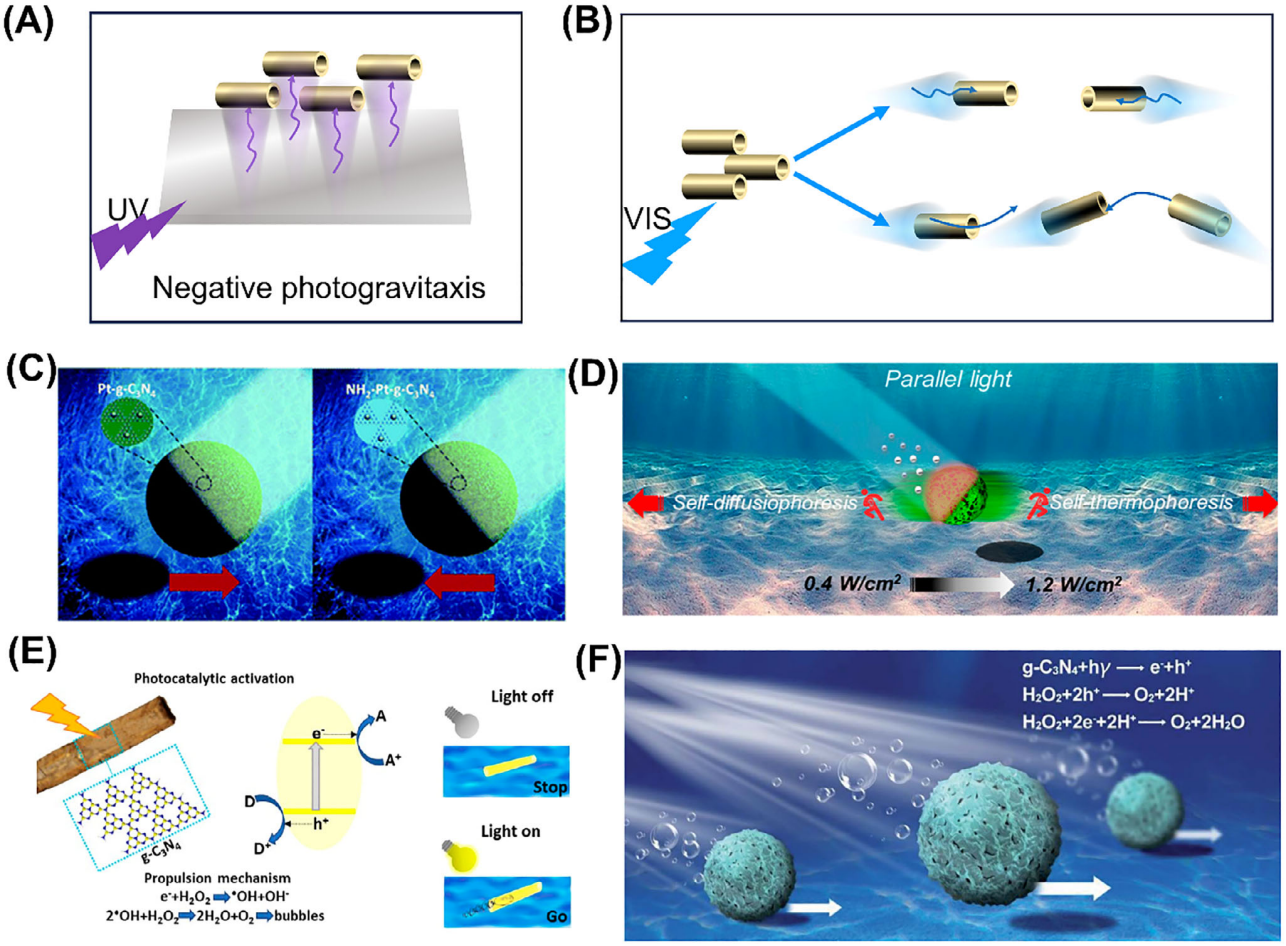
Photocatalytic propulsion mechanism of g‐C_3_N_4_ microrobots. (A) Upon UV illumination, g‐C_3_N_4_ microrobots move upward, exhibiting negative photogravitaxis. (B) Under visible light, they display both 2D and 3D motion behaviors. (A,B) Reproduced with permission [2]. under the CC BY 4.0 license. Copyright 2025, The Authors. (C) Pt–g‐C_3_N_4_ microrobots exhibit positive phototaxis, whereas NH_2_‐Pt‐g‐C_3_N_4_ microrobots display negative phototactic behavior. Reproduced with permission [36]. Copyright 2017, The Royal Society of Chemistry. (D) Scheme of self‐diffusiophoresis and self‐thermophoresis mechanisms with varying light intensities. Reproduced with permission [37]. Copyright 2021, National Academy of Sciences. (E) Schematic of the photocatalytic process and bubble propulsion mechanism of light‐controlled g‐C_3_N_4_‐based tubular microrobots in H_2_O_2_ solution. Reproduced with permission [34]. Copyright 2018, American Chemical Society. (F) g‐C_3_N_4_@CMS microrobots exhibit bubble propulsion under visible light irradiation and H_2_O_2_. Reproduced with permission [31]. from the Royal Society of Chemistry. Copyright 2022, The Authors.

The propulsion of Fe_3_O_4_/3D porous foam‐like C_3_N_4_ (f‐C_3_N_4_) microrobots can also be explained by the self‐diffusiophoretic mechanism [25]. Under visible light irradiation in the presence of 0.2% H_2_O_2_, the microrobots exhibited an effective diffusion coefficient of 1.77 µm^2^ s^−^^1^. Their propulsion behavior is attributed to the irregular morphology of Fe_3_O_4_/f‐C_3_N_4_ and the non‐uniform distribution of Fe_3_O_4_, which create asymmetric concentrations of molecules, ions, and reactive oxygen species around the microrobots. The resulting chemical asymmetry breaks the symmetry of the surrounding fluid flow, leading to self‐diffusiophoretic propulsion.

Another example of self‐diffusiophoretic propulsion is provided by the Pt–g‐C_3_N_4_ spherical microrobots (Figure 3C) [36]. These microrobots are light‐responsive, remaining stationary in water without any added chemical fuel but becoming motile under white light. Depending on surface modification, they can display either positive or negative phototaxis. Specifically, Pt–g‐C_3_N_4_ microrobots carried a negative charge in water and moved toward the light source (positive phototaxis) at a speed of about 14 µm/s. As the light intensity increased, their speed increased accordingly and reached a maximum of 23 µm/s. After surface modification with an amine‐functionalized thiol, they became positively charged and displayed negative phototaxis, moving away from the light at ∼12 µm/s. This speed reduction may be attributed to the decreased catalytic activity of platinum after modification. The motion originates from a self‐diffusiophoretic mechanism in which photocatalytic water oxidation and oxygen reduction generate reactive species (e.g., H^+^, •OH, •O_2_^−^), thereby forming chemical gradients that drive propulsion.

In addition, the poly(heptazine imide) (PHI)─Pt, PHI─Au, and PHI─SiO_2_ microswimmers prepared by Sridhar et al. are also primarily propelled by self‐diffusiophoresis [39]. Under 365 nm illumination, they exhibited self‐propulsion in water with speeds of 10.4 µm/s, 9.1 µm/s, and 8.6 µm/s, respectively. PHI─Pt showed the highest speed (20.4 µm/s) in 5 vol% methanol, while PHI─Au reached 24.6 µm/s in 0.5 vol% H_2_O_2_. Overall, PHI─Pt performed better in water and methanol, whereas PHI─Au and PHI─SiO_2_ were faster in H_2_O_2_. Self‐diffusiophoresis is the dominant propulsion mechanism under all conditions, while self‐electrophoresis, resulting from interfacial charge separation, provides an additional contribution in methanol and H_2_O_2_, especially in PHI─Pt and PHI─Au systems. Remarkably, some microswimmers exhibited ballistic swimming for over 30 min in the dark after just 30 s of light. This phenomenon, known as a “solar battery swimming,” is attributed to the efficient reactions of H_2_O_2_ as fuel and PHI's ability to store and slowly release photogenerated electrons.

Carbon nitride/polypyrrole nanoparticle (C_3_N_4_/PPyNP)‐based microrobots show biomimetic individual and collective bidirectional phototaxis, which results from the competition between self‐diffusiophoresis and self‐thermophoresis (Figure 3D) [37]. Upon weak light irradiation (0.4 W/cm^2^), C_3_N_4_ acts as a photocatalyst that generates and separates electron–hole pairs, where the electrons reduce oxygen to produce superoxide radicals (•O_2_^−^) while the holes oxidize water to form protons (H^+^) and hydroxyl radicals (•OH). These ionic species diffuse at different rates, with protons diffusing the fastest, thus creating a local electric field. Owing to their negatively charged surfaces, the microrobots are propelled toward the light source through self‐diffusiophoresis. However, under high‐intensity light (1.2 W/cm^2^), the photothermal effect of the PPyNP component becomes dominant. This induces self‐thermophoresis, causing the microrobots to exhibit negative phototaxis and move away from the light. This work lays a foundation for intelligent light‐responsive systems by demonstrating light‐intensity‐dependent motion and collective responses in a fuel‐free environment.

Bubble propulsion has also been reported in several studies. For example, our group developed another type of g‐C_3_N_4_‐based tubular microrobot driven by this mechanism (Figure 3E) [34]. Photogenerated electrons interact with H_2_O_2_ to generate •OH, which then further reacts with H_2_O_2_ to produce H_2_O and O_2_. The O_2_ bubbles grow inside the tube and are ejected through the opening, propelling the microrobots in the opposite direction with a maximum speed of 72 µm/s at 20 wt% H_2_O_2_. Tubular g‐C_3_N_4_‐Fe_3_O_4_@KF microrobots are propelled by the bubble recoil mechanism in a 30 wt% H_2_O_2_ and 1% SDS solution with 420 nm light [29]. Electrons and holes generated on the microrobot surface catalyze H_2_O_2_ decomposition to form O_2_ bubbles, which are expelled and drive the microrobots through recoil. The Fe component also undergoes redox reactions with H_2_O_2_, providing additional oxygen for bubble formation. Pt/Pd@g‐C_3_N_4_@CSs can also be propelled by bubble recoil, reaching velocities of 14.5 ± 3.4 µm/s in 7.5% H_2_O_2_ solution under λ > 400 nm light irradiation [32]. g‐C_3_N_4_@CMS microrobots can acquire bubble propulsion in 30 wt% H_2_O_2_, reaching a maximum speed of approximately 167.97 µm/s under 420 nm light illumination (Figure 3F) [31]. These charge carriers react with H_2_O_2_ on the illuminated side to produce O_2_, which forms bubbles that are then released asymmetrically, driving the microrobots forward through recoil.

The Janus Fe/C_3_N_4_ microrobots are mainly driven by light‐induced self‐electrophoresis, while oxygen generation further enhances propulsion [38]. g‐C_3_N_4_ produces electron–hole pairs under 532 nm laser illumination. Subsequently, these electrons transfer to the Fe layer and participate in redox reactions while the holes oxidize water. These processes create a local electric field that drives motion. The addition of H_2_O_2_ increases propulsion by reacting with accumulated electrons to generate O_2_. When both H_2_O_2_ and Cr(VI) are present, the Cr(VI)/H_2_O_2_ redox reaction produces additional O_2_ during Cr(VI) reduction, further accelerating microrobot motion. As the Cr(VI) concentration increases from 0 ppm to 5 ppm, the diffusion coefficient (D) rises from 12 × 10^6^ nm^2^/s to 44 × 10^6^ nm^2^/s.

In summary, the propulsion of g‐C_3_N_4_‐based microrobots depends on their structural design and the surrounding chemical environment. Depending on these factors, motion can be driven by self‐diffusiophoresis, self‐electrophoresis, bubble recoil, or a combination of these mechanisms. Self‐diffusiophoresis and self‐electrophoresis are typically based on asymmetric distributions of ions or charges generated during photocatalytic reactions. These mechanisms allow microrobots to move under low fuel concentrations and, in some cases, even in fuel‐free conditions. In contrast, bubble propulsion relies on rapid gas generation and bubble ejection, usually requires high concentrations of chemical fuel, and often results in higher propulsion speeds. Through these mechanisms, g‐C_3_N_4_‐based microrobots can achieve light‐controlled motion, directional migration, phototaxis, and collective behaviors. This versatility makes them promising platforms for the development of intelligent and light‐responsive microsystems.

## 4 Applications, Challenges, and Prospects of g‐C_3_N_4_ Microrobots in Water Purification

### 4.1 g‐C_3_N_4_ Microrobots in Water Purification

#### 4.1.1 Applications of g‐C_3_N_4_ Microrobots in Water Purification

g‐C_3_N_4_‐based materials remove pollutants from water primarily through photocatalytically generated reactive species such as •O_2_^−^, •OH, ^1^O_2_, and holes [40]. Under light irradiation, electrons in the valence band (VB) of g‐C_3_N_4_ are promoted to the conduction band (CB) while holes remain in the VB. These charge carriers then react with O_2_, H_2_O, or H_2_O_2_ to generate reactive species that degrade pollutant molecules. The following sections describe in detail the applications of g‐C_3_N_4_‐based microrobots in water pollutant removal.

Our group used g‐C_3_N_4_ microrobots (microtubes) for the degradation of tetracycline (TC) in water, reaching 77% removal efficiency within 90 min (Figure 4A) [2]. A further investigation was carried out to explore the degradation mechanism of TC (Figure 4B). UV–vis absorption and XPS analysis indicated that the valence band (VB) was located at +2.08 eV, whereas the conduction band (CB) lay at −0.52 eV. Because the VB is positioned above the OH^−^/•OH redox potential (+1.99 eV), holes (h^+^) can oxidize OH^−^ to form •OH radicals. In contrast, the CB is more negative than the O_2_/•O_2_^−^ redox potential (−0.33 eV), allowing O_2_ to accept electrons and form •O_2_^−^ (Figure 4B). The generated •O_2_^−^, h^+^, and •OH subsequently react with TC, resulting in its degradation. Selective scavenging experiments targeting these species further supported this mechanism and identified •O_2_^−^ as the dominant reactive species. Furthermore, electron paramagnetic resonance (EPR) experiments confirmed that the microrobots generated both •O_2_^−^ and •OH under light irradiation (Figure 4C). The g‐C_3_N_4_/Ag Janus microrobots developed by our group could also degrade tetracycline with an efficiency of 88% within 90 min [33]. The degradation was attributed to the attack of •O_2_^−^, singlet oxygen (^1^O_2_), h^+^, and •OH on the tetracycline structure (Figure 4D). Among them, the g‐C_3_N_4_/Ag Janus microrobots exhibit negative photogravitaxis under light irradiation, which facilitates tetracycline degradation. Moreover, selective scavenging experiments suggest that •O_2_^−^ is the main reactive species during the degradation process. It contributes to degradation through direct oxidation of tetracycline and indirectly via conversion to ^1^O_2_ (Figure 4E). In contrast, h^+^ and •OH make only limited contributions. Meanwhile, experiments conducted under N_2_‐saturated conditions confirmed the critical role of photogenerated electrons. EPR analysis further confirmed the presence of •O_2_^−^, ^1^O_2_, and •OH. Additionally, HPLC–MS analysis confirmed the presence of several degradation intermediates, including oxidized tetracycline (m/z 418.130, C_22_H_23_N_2_O_9_^+^), a dehydrated form of tetracycline (m/z 416.133, C_22_H_22_NO_9_^+^), a hydroxylated derivative (m/z 434.166), and two unidentified degradation products (m/z 418.170 and 416.157) (Figure 4F). These two studies demonstrate the effective application of g‐C_3_N_4_‐based microrobots for photocatalytic tetracycline degradation in water and clarify the underlying reaction mechanism. Through band structure analysis, identification of degradation intermediates, and confirmation of •O_2_^−^ as the major reactive species, this work reveals the potential of microrobots for environmental remediation.

**FIGURE 4:**
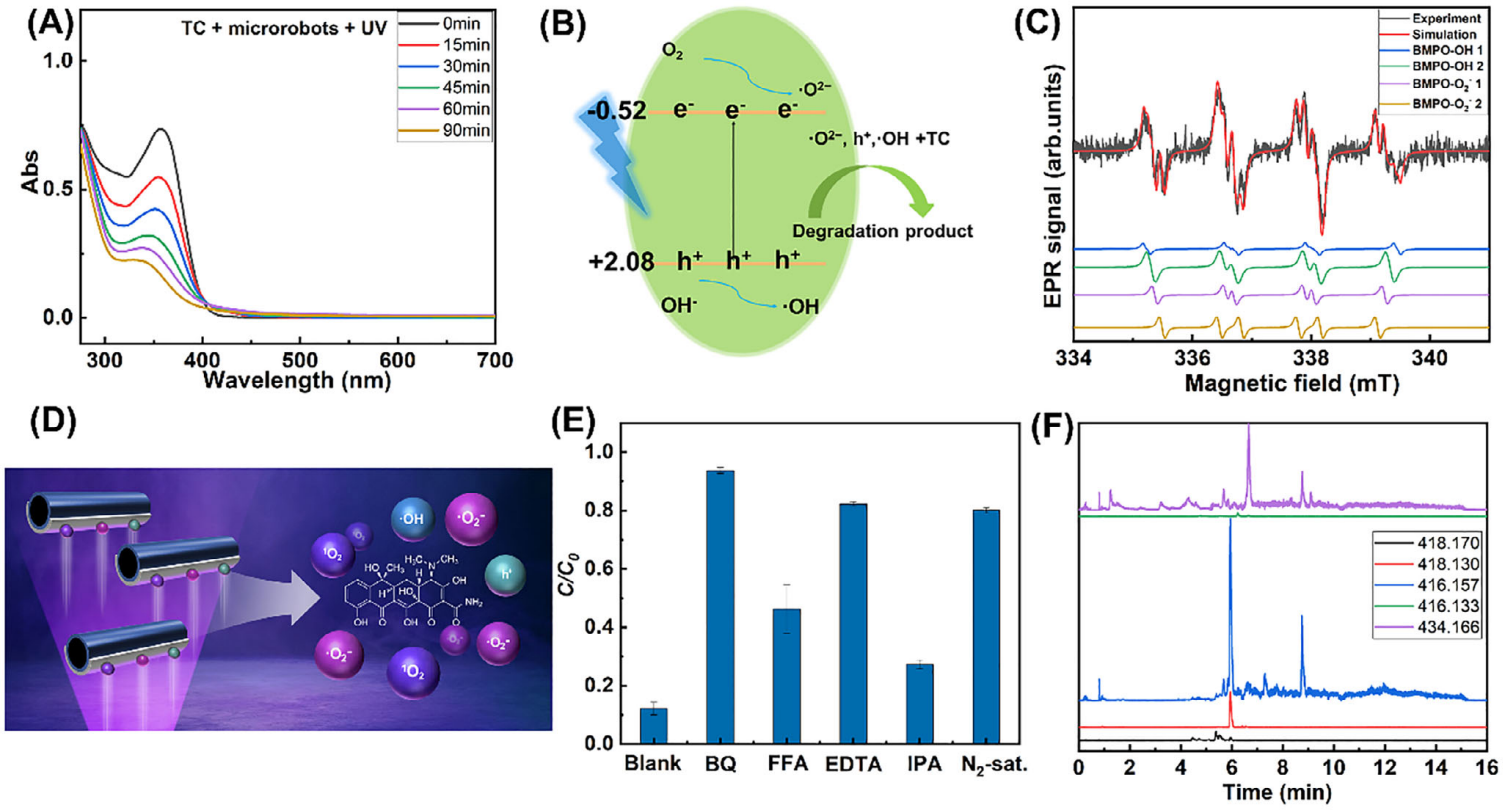
Photocatalytic tetracycline degradation using g‐C_3_N_4_ microrobots (A–C) and g‐C_3_N_4_/Ag Janus microrobots (D–F) [2, 33]. (A) Evolution of UV–vis absorption spectra during photocatalytic degradation of tetracycline. (B) Schematic mechanism of tetracycline photodegradation by microrobots. (C) EPR spectra and simulated fitting curves for •O_2_^−^ and •OH radicals detected under light, with BMPO used as the trapping agent. (A–C) Reproduced with permission [2]. under the CC BY 4.0 license. Copyright 2025, The Authors. (D) Tetracycline degradation by g‐C_3_N_4_/Ag Janus microrobots under light irradiation, where the microrobots exhibit negative photogravitaxis and generate reactive species (•O_2_^−^, ^1^O_2_, h^+^, and •OH). (E) Effect of radical scavengers and N_2_ atmosphere on tetracycline removal efficiency. (F) Extracted ion chromatograms (EICs) showing photocatalytic degradation intermediates. Identified compounds include an oxidized species (m/z 418.130, TCKOx), a dehydrated product (m/z 416.133, TCKdehyd), and a hydroxylated derivative (m/z 434.166). Two additional unknown intermediates are observed at m/z 418.170 and 416.157. (D–F) Reproduced with permission [33]. under the CC BY 4.0 license. Copyright 2026, The Authors.

Fe_3_O_4_/f‐C_3_N_4_ microrobots were also employed to degrade tetracycline, achieving a high degradation efficiency of 96.4% within 90 min under 2% H_2_O_2_ and a rotating magnetic field [25]. Notably, under the same experimental conditions, Fe_3_O_4_ or f‐C_3_N_4_ alone exhibited significantly lower removal efficiencies, indicating that the Fe_3_O_4_/f‐C_3_N_4_ structure significantly enhances pollutant degradation. Fe_3_O_4_/f‐C_3_N_4_ degraded tetracycline through visible‐light photocatalysis and Fenton‐like reactions, producing reactive species such as •OH and •O_2_^−^. These species and photogenerated holes decomposed tetracycline into intermediate products, and the proposed degradation pathways are illustrated in Figure 5A. These intermediates were subsequently transformed into small molecules, water, and carbon dioxide. The porous structure of f‐C_3_N_4_ provides a large surface area while microrobot motion enhances mass transfer, contributing to the overall degradation process.

**FIGURE 5:**
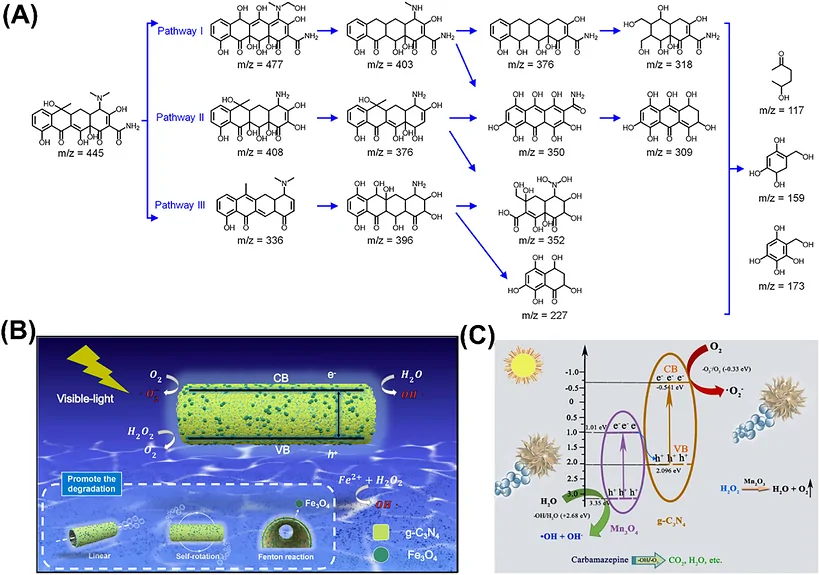
Schematic illustrations of representative mechanisms for pollutant degradation using g‐C_3_N_4_‐based microrobots under visible light. (A) Possible photocatalytic degradation pathways of tetracycline. Reproduced with permission [25]. Copyright 2022, American Chemical Society. (B) Visible‐light‐driven degradation of Rhodamine B by g‐C_3_N_4_/Fe_3_O_4_ microrobots, facilitated by self‐propulsion and self‐rotation. Reproduced with permission [29]. Copyright 2022, Elsevier. (C) Oxidative degradation of carbamazepine by Mn_3_O_4_/C_3_N_4_ Janus microrobots through free radical generation. Reproduced with permission [41]. under the CC BY 4.0 license. Copyright 2024, The Authors.

g‐C_3_N_4_/Fe_3_O_4_@KF microrobots have been used to degrade Rhodamine B under visible light with 3% H_2_O_2_ [29]. A 10 mg/L Rhodamine B solution was nearly fully degraded within 60 min. The excellent degradation performance is attributed to the unique combination of self‐propulsion and self‐rotation, which enhances micro‐mixing and thereby improves mass transfer, further facilitating interactions between the microrobots and pollutants (Figure 5B). This dynamic behavior significantly improves the photocatalytic degradation efficiency compared to static systems. g‐C_3_N_4_@CSs have also been utilized for the degradation of Rhodamine B under visible light and magnetic stirring [32]. They removed 51% of Rhodamine B, with an average removal rate that was three times higher than that of pure g‐C_3_N_4_ powder. This improved rate is attributed to the increased light absorption of g‐C_3_N_4_@CSs, the enhanced charge separation provided by the lignin‐based carbon spheres, and heterojunction formation between g‐C_3_N_4_ and the carbon spheres. Another microrobot system for Rhodamine B degradation is based on g‐C_3_N_4_@CMS [31]. These microrobots degraded 95.88% of the pollutant within 60 min under visible light with 3 wt% H_2_O_2_. In contrast, the same system exhibited only 31.10% efficiency in the dark, indicating limited degradation without light. Under visible light without H_2_O_2_, the degradation efficiency was 55.48%, markedly lower than that observed when H_2_O_2_ was present. Without H_2_O_2_ as a fuel, the microrobots are unable to achieve self‐propulsion, which significantly limits their degradation capability. Therefore, the excellent degradation performance can be attributed to two key factors. First, H_2_O_2_ serves as a chemical fuel that drives the autonomous motion of the micromotors, thereby enhancing fluid mixing and increasing contact between the micromotors and the pollutants. Second, the breakdown of H_2_O_2_ produces radicals, which further accelerate the photocatalytic degradation process. These findings demonstrate a synergistic effect between photocatalysis and self‐propulsion in enhancing microrobot‐based pollutant removal, offering valuable insights for the future development of microrobotic systems for environmental remediation.

Fe/C_3_N_4_ microrobots combined with Cr(VI)/H_2_O_2_ were applied to degrade sulfamethoxazole (SMX) [38], an emerging contaminant commonly found in pharmaceutical residues. A 1 ppm SMX solution was completely degraded within 8 min. Scavenging experiments revealed that O_2_·^−^, ·OH, and ^1^O_2_ were the dominant reactive species. Importantly, the system does not rely on noble metals such as Pt or Au as electron receptors, thereby improving its economic feasibility. The study demonstrates the potential of this system for the simultaneous reduction of Cr(VI) and degradation of organic pollutants in contaminated water.

Another study reported 3D‐printed Fe_3_O_4_/C_3_N_4_ robots for picric acid (PA) degradation [26]. These robots removed 38% of PA within 10 min under visible‐light irradiation. C_3_N_4_ acted as the photocatalyst, producing superoxide radicals and holes, which served as the main active species for PA degradation, while the chitosan hydrogel functioned as an adsorbent. The enhanced degradation is attributed to the synergistic effect of adsorption by chitosan and photocatalysis by C_3_N_4_. Because heterogeneous photocatalysis primarily occurs at molecular interfaces, it is crucial that pollutants can effectively adsorb onto the photocatalyst surface. Yang et al. applied Mn_3_O_4_/C_3_N_4_ Janus microrobots to degrade the widely used antibiotic carbamazepine (CBZ) (Figure 5C) [41]. Within 300 min, the Mn_3_O_4_/C_3_N_4_‐JMC achieved 79.3% CBZ degradation, markedly higher than that of Mn_3_O_4_/C_3_N_4_ (55.5%), g‐C_3_N_4_, C_3_N_4_/C, or H_2_O_2_ alone. The degradation performance can be attributed to two factors. First, Mn_3_O_4_ rapidly decomposes H_2_O_2_ to generate O_2_ bubbles, which enhance mass transfer and thereby increase the interaction between pollutants and the microrobots. Second, the Z‐scheme heterojunction between Mn_3_O_4_ and g‐C_3_N_4_ promotes charge separation, facilitating the reaction of photogenerated electrons and holes with OH^−^ and O_2_ to generate highly oxidative radicals, which then degrade carbamazepine.

Although most studies have emphasized the photocatalytic activity of g‐C_3_N_4_, our group has also reported work highlighting its interactions with pollutants [34]. Tubular g‐C_3_N_4_ micromotors removed 50% of Cu^2^^+^ ions from a 15 ppm solution and achieved complete removal at 1 ppm when combined with 5 wt% H_2_O_2_ under visible light. This efficient removal can be ascribed to the strong affinity of Cu^2^^+^ ions for the g‐C_3_N_4_ surface through complexation with nitrogen‐ and carbon‐containing functional groups, Lewis acid–base interactions with the delocalized π electrons of the triazine rings, and electrostatic interactions with the negatively charged g‐C_3_N_4_ surface. Autonomous bubble‐propelled motion further enhanced micromixing and mass transfer. ATR‐FTIR and XPS analyses further confirmed these interactions, revealing Cu^2^^+^ complexation with surface functional groups and electron transfer from g‐C_3_N_4_ nitrogen atoms to the metal ions. The lower removal efficiency at 15 ppm primarily resulted from the saturation of the available adsorption sites, as Cu^2^^+^ adsorption reached a plateau at around 10 ppm.

#### 4.1.2 Challenges and Prospects of g‐C_3_N_4_ Microrobots in Water Purification

g‐C_3_N_4_‐based microrobots have shown great promise for water purification. While the photocatalytic degradation of pollutants is their primary and most effective application, their application in heavy metal adsorption has also been explored. However, several challenges remain before their practical deployment can be realized. Most research is limited to laboratory conditions and does not consider real water environments. Such environments contain salts, suspended particles, natural organic matter, and microorganisms, which may interfere with microrobot motion and performance. Another challenge is that the concentration of H_2_O_2_ in open water is difficult to control and maintain, which limits long‐term microrobot motion. In addition, some studies have used high concentrations of H_2_O_2_ as fuel. This may create strongly oxidative conditions, potentially harming aquatic organisms and disrupting the ecological balance. The limited penetration of visible and UV light restricts the performance of light‐driven microrobots in turbid or deep waters. Although g‐C_3_N_4_ is often considered environmentally friendly, the long‐term ecological effects of microrobot deployment should also be considered, particularly when the microrobots become inactive or reach the end of their service life and cannot be effectively recovered. Finally, research is still mostly at the laboratory stage, and large‐scale production has not yet been well developed.

To solve these challenges, future research should involve testing under realistic water conditions and developing surface modifications to reduce interference from organic matter and microorganisms. Fuel‐free and bio‐derived propulsion strategies should be further investigated to address current limitations in microrobot propulsion. Future studies should also consider toxicity, feasibility, and environmental safety in practical applications. Light penetration limitations may be overcome by enhancing light absorption through composite heterojunctions with carbon materials, oxides, or other components, and by developing hybrid propulsion systems. Environmental safety can be improved through systematic toxicity evaluation, the design of biodegradable microrobots, and effective strategies for their recovery and removal after use. In addition, g‐C_3_N_4_‐based materials could be immobilized in a membrane‐based flow‐through water treatment system, where the active materials are retained within the system while water passes through. By controlling the flow rate and residence time, sufficient contact between the contaminated water and the active materials can be maintained for photocatalytic treatment. Meanwhile, the development of low‐cost synthesis and large‐scale production technologies is important for the practical application of these systems. These strategies can facilitate safe, efficient, and sustainable applications of g‐C_3_N_4_‐based microrobots in real‐world environmental remediation.

Besides antibiotics and dyes, g‐C_3_N_4_‐based microrobots may also be promising for treating other challenging or emerging pollutants, such as pharmaceutical and phenolic micropollutants, endocrine disruptors, and pesticides [40, 42]. These pollutants have already been explored using conventional g‐C_3_N_4_‐based photocatalysts. Under light irradiation, g‐C_3_N_4_ generates reactive species such as •O_2_^−^, •OH, and h^+^, which can react with pollutants, thereby degrading them. For microrobots, autonomous motion can further improve mixing and increase contact between the photocatalyst and pollutants, thereby enhancing mass transfer and pollutant removal. Therefore, these pollutants could be important targets for future studies of g‐C_3_N_4_‐based microrobots.

### 4.2 g‐C_3_N_4_ Microrobots in Biomedical Applications

#### 4.2.1 Applications of g‐C_3_N_4_ Microrobots in Biomedical Scenarios

g‐C_3_N_4_‐based microrobots are increasingly being explored for biomedical applications due to their unique photoactive properties, high stability, and good biocompatibility. Under light irradiation, they produce reactive oxygen species (ROS) that can disrupt bacterial membranes and damage intracellular components. In addition, g‐C_3_N_4_ derivatives with structural nanoporosity allow high‐capacity drug loading and light‐controlled release, providing opportunities for targeted cancer therapy and theranostic applications in hypoxic environments.

Guo et al. developed liquid metal (LM)‐based photocatalytic microrobots, namely Ga–g‐C_3_N_4_@Pt microrobots, which were used for photocatalytic antibacterial therapy under UV illumination in low‐concentration H_2_O_2_ (Figure 6A) [43]. These microrobots generated reactive oxygen species (ROS), particularly singlet oxygen (^1^O_2_), and simultaneously released Ga^3^^+^ ions. Both factors contributed to efficient bacterial killing, achieving a 99% reduction in *E. coli*. Moreover, their autonomous motion enhanced the local diffusion of antibacterial species, resulting in increased contact with bacteria. In vivo studies in infected wound models confirmed that the microrobots accelerated wound healing by promoting epithelial regeneration and collagen deposition, highlighting their potential as active platforms for antibacterial therapy and tissue repair.

**FIGURE 6:**
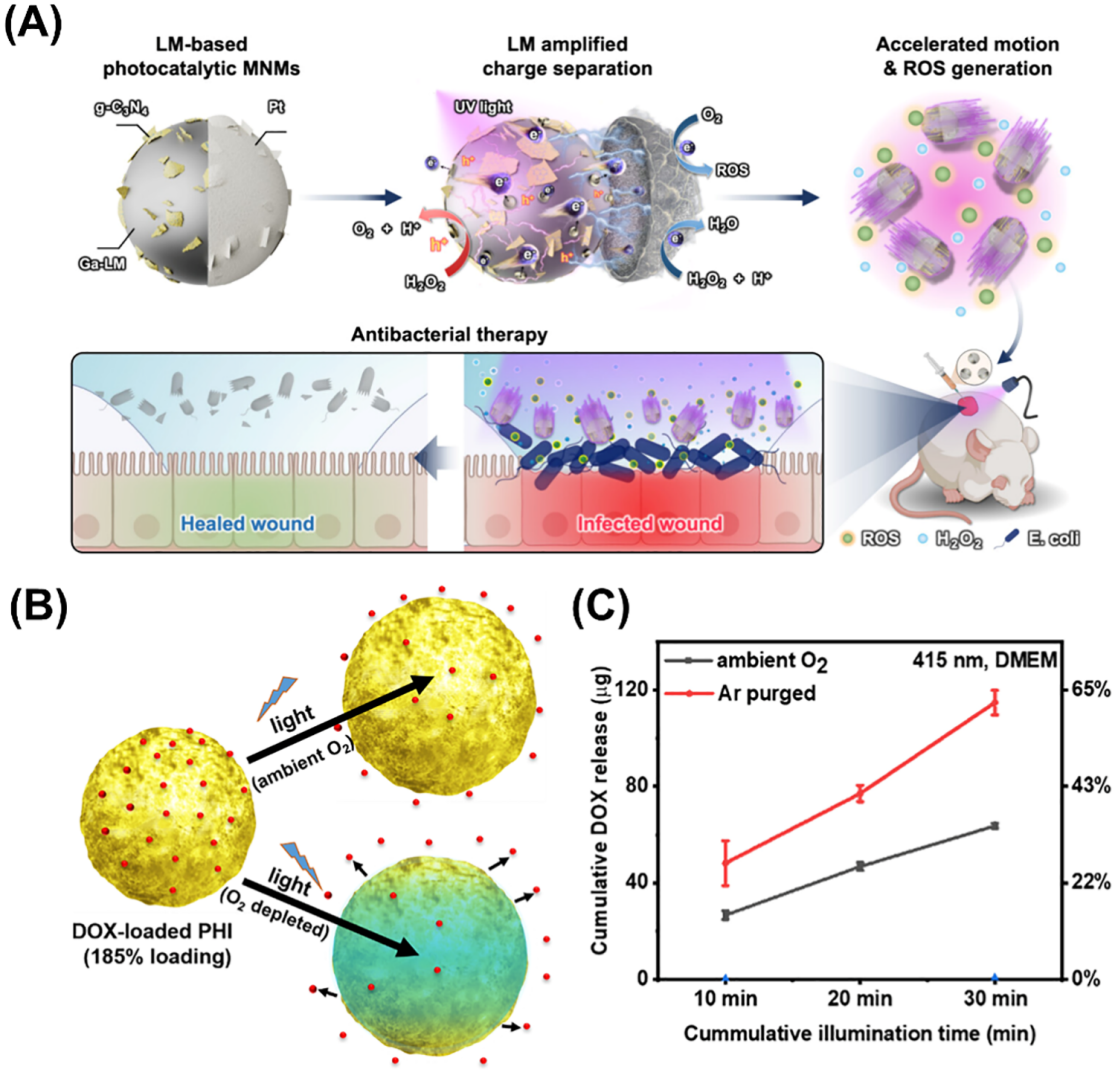
g‐C_3_N_4_‐based microrobots for biomedical applications. (A) Ga–g‐C_3_N_4_@Pt microrobots demonstrate light‐driven motion and antibacterial therapy by generating reactive oxygen species. Reproduced with permission [43]. Copyright 2025, American Chemical Society. (B) Illustration of PHI microrobots loaded with doxorubicin (DOX) and its light‐triggered release under ambient oxygen conditions and oxygen‐deficient conditions. (C) Time‐dependent release of DOX in DMEM under 415 nm light. The supernatant was removed and measured at each time point in both ambient air (black) and argon‐purged (red) environments. (B,C) Reproduced with permission [27]. Copyright 2022, AAAS.

Sridhar et al. synthesized 2D poly(heptazine imide) (PHI) carbon nitride microrobots, a crystalline derivative of g‐C_3_N_4_ [27]. These microrobots exhibited self‐propulsion in various biological media, demonstrated biocompatibility, and enabled light‐controlled release of the cancer drug doxorubicin. Cytotoxicity tests with NIH 3T3, SKBR3, and HT‐29 cells showed that all three maintained high viability even at a microrobot concentration of 30 µg/mL. Owing to their textural and structural nanoporosity, these PHI microrobots achieved a doxorubicin loading efficiency of 185%, much higher than the 20–70% typically reported for other systems (Figure 6B,C). When exposed to 415 nm blue light, approximately 35% of the drug was released from PHI microswimmers within 30 min under ambient oxygen conditions, whereas under oxygen‐deficient conditions, the release increased to around 62%, which was attributed to the light‐induced photocharging effect of PHI. Prolonged illumination beyond 60 min under ambient conditions led to a reduction in DOX release, indicating partial light‐triggered degradation. This oxygen‐sensitive, light‐triggered release behavior highlights the potential of PHI microrobots for controlled drug delivery and theranostic applications, particularly in hypoxic tumornments.

#### 4.2.2 Challenges and Prospects of g‐C_3_N_4_ Microrobots in Biomedical Applications

Based on these reports, it is evident that g‐C_3_N_4_‐based microrobots have been primarily explored for environmental remediation, especially in the photocatalytic degradation of pollutants; however, only limited studies have investigated their biomedical potential. This is mainly because g‐C_3_N_4_ exhibits strong photocatalytic activity and abundant surface functional groups, making it highly effective for decomposing organic pollutants and adsorbing heavy metals in water [44]. In contrast, biomedical applications involve experiments in cellular and animal models, which require strict evaluation of biosafety and ethical considerations, making such studies more complex, time‐consuming, and costly [45, 46].

A major challenge is the precise regulation of ROS generation. While ROS are essential for antibacterial and anticancer applications, uncontrolled ROS could damage healthy cells, and their distribution and release become even more complicated during microrobot motion. Moreover, bulk g‐C_3_N_4_ has poor solubility and limited biodegradability, which restricts its direct biomedical use [47]. Although various modified forms of g‐C_3_N_4_ have shown improved water dispersibility and stability, their high stability still hinders biodegradation in vivo [48]. The motion of microrobots further increases uncertainty in biodistribution, retention, and clearance, making parameters such as renal clearance efficiency and tissue distribution critical for evaluation [49]. Despite encouraging preliminary results [50], systematic studies on long‐term metabolism, immune responses, and comprehensive biosafety evaluation are still lacking. Due to their dynamic motion and complex surface modifications, microrobots may exhibit distinct biological interactions, highlighting the need for more thorough investigation [50]. Another limitation lies in the optical properties of g‐C_3_N_4_. Because its absorption spectrum is mainly confined to wavelengths below 460 nm, its utilization of visible light is limited, restricting applications in deep‐tissue imaging and photodynamic therapy [51].

In the future, several strategies may help overcome these challenges. First, targeted ROS release could be achieved by controlling the motion of microrobots, while element doping and bandgap engineering can be employed to regulate ROS yield, and magnetic control can also be introduced to achieve more precise manipulation. Second, poor solubility and limited degradability may be addressed by hybridizing g‐C_3_N_4_ with degradable polymers or designing partially degradable microrobots. Most importantly, systematic evaluations of in vivo biosafety, renal clearance efficiency, long‐term metabolism, and immune responses are urgently needed. To address the limited absorption range of g‐C_3_N_4_, which is primarily restricted to wavelengths below 460 nm, several strategies can be considered, including bandgap tuning through nonmetal doping, coupling with upconversion nanoparticles, and photosensitization. For example, Liu et al. modified protonated g‐C_3_N_4_ with protoporphyrin, and the resulting material exhibited activity under either visible–near‐infrared light (Vis–NIR, λ > 420 nm) or near‐infrared light (NIR, λ > 780 nm) [52]. These approaches may improve deep‐tissue light penetration and expand biomedical applicability. Furthermore, optical fibers can be used to deliver light directly to otherwise inaccessible internal tissues, including through endoscopic or catheter‐based approaches [53]. Recent advances in g‐C_3_N_4_‐based biomedical materials may also provide useful design strategies for the future development of g‐C_3_N_4_‐based microrobots in biomedical applications. For example, potassium/cyano group‐functionalized g‐C_3_N_4_ has recently been reported to exhibit enhanced charge separation and ROS generation under NIR‐II irradiation, supporting its application in multiphoton photodynamic therapy [54]. Such materials engineering strategies may help extend the photoresponse of g‐C_3_N_4_‐based microrobots and facilitate their future in vivo applications.

## 5 Summary, Challenges, and Future Perspectives

Graphitic carbon nitride (g‐C_3_N_4_) is a stable, metal‐free, environmentally friendly, and low‐cost visible‐light photocatalyst that has been widely applied in environmental remediation and has also attracted growing attention in biomedical research. In recent years, researchers have begun to employ g‐C_3_N_4_ as a functional material for microrobots. However, compared with commonly used photocatalytic materials such as TiO_2_ and ZnO, research on g‐C_3_N_4_‐based microrobots is still at an early stage. Photocatalytic propulsion based on fuel or water decomposition requires specific structural designs, and most reported studies have focused on tubular or spherical shapes, which remain challenging to fabricate. Although simple methods such as direct calcination and ultrasonication can produce sheet‐like g‐C_3_N_4_, these structures often need additional components to achieve effective propulsion or targeted applications. Therefore, developing simple and efficient methods to synthesize g‐C_3_N_4_ with controllable morphologies is crucial for advancing microrobotic applications. Moreover, a deeper understanding of the propulsion mechanisms of g‐C_3_N_4_‐based microrobots is essential to establish a theoretical foundation for future applications.

Another major challenge is to enhance the generation and utilization efficiency of photogenerated charge carriers to improve photocatalytic activity and propulsion performance. Constructing composite heterojunctions with carbon materials, oxides, or other semiconductors can suppress electron–hole recombination, broaden the light absorption range, and facilitate charge transfer. In addition, elemental doping can tune the electronic structure, bandgap, and surface activity of g‐C_3_N_4_, further optimizing its photocatalytic behavior.

g‐C_3_N_4_‐based microrobots have shown great potential in environmental remediation. However, several problems still need to be considered before practical application, including limited motion controllability, challenges in fuel delivery, shallow penetration of visible and UV light, limited evaluation of environmental safety under realistic conditions, and difficulties in large‐scale implementation. Future research should focus on developing fuel‐free and bio‐derived propulsion strategies, enhancing light absorption and utilization, conducting more systematic biosafety evaluations, and designing biodegradable microrobots. Experiments under realistic water conditions, along with the development of low‐cost and scalable synthesis methods, will be essential for real‐world applications. Moreover, the biomedical potential of g‐C_3_N_4_‐based microrobots is beginning to attract the interest of researchers.

Although biomedical studies on g‐C_3_N_4_‐based microrobots remain limited, their photoactive properties, high stability, and biocompatibility make them promising candidates. Future studies should concentrate on improving their biosafety, biodegradability, light penetration, and controllability, as well as conducting comprehensive in vivo evaluations.

In summary, g‐C_3_N_4_ is a metal‐free and sustainable photocatalyst with strong potential for light‐driven microrobots. Further progress will rely on combining materials design, photophysics, and environmental and biomedical engineering to move these systems from laboratory studies toward practical applications in environmental remediation and medicine.

## Author Contributions

**Yunhuan Yuan**: writing – original draft, Writing – review and editing. **Martin Pumera**: writing – review and editing, writing – original draft, resources, supervision, project administration.

## Conflicts of Interest

M.P. joined the Scientific Advisory Board of Selene Nanorobotics Inc, USA, after the completion and initial submission of this work and holds stock options in the company. M.P. cofounded Spintuo s.r.o., Czech Republic, after the completion and initial submission of this work. These relationships arose only after the submission of the manuscript and had no role in the conception, design, execution, analysis, interpretation, or preparation of the article. M.P. discloses these interests in the interest of full transparency. The other authors declare no conflicts of interest.

## Data Availability

The data that support the findings of this study are available from the corresponding author upon reasonable request.
